# P-749. Increased Length of Stay and Higher Healthcare Costs Associated with Fournier Gangrene Compared to Non-Perineal Necrotizing Soft Tissue Infections: A Retrospective Analysis of the National Inpatient Sample (2016–2020)

**DOI:** 10.1093/ofid/ofaf695.960

**Published:** 2026-01-11

**Authors:** Hayato Mitaka, Kristen McQuerry, Kelsey Karnik, Alexandre Marra, Yuji Yamada, Takaaki Kobayashi

**Affiliations:** University of Colorado, Aurora, CO; University of Kentucky, Lexington, Kentucky; University of Kentucky, Lexington, Kentucky; University of Iowa Hospital and Clinics, iowa city, Iowa; Icahn School of Medicine at Mount Sinai, New York, New York; University of Kentucky, Lexington, Kentucky

## Abstract

**Background:**

Fournier gangrene (FG) is a necrotizing soft tissue infection (NSTI) of the perineum. Recent retrospective studies from quaternary centers suggest improved outcomes and a potentially less aggressive clinical course for FG than non-perineal NSTIs. However, comprehensive nationwide data remain limited.

**Methods:**

This retrospective cohort study analyzed the National Inpatient Sample (2016–2020) to compare outcomes between FG and non-perineal NSTIs. Adult patients undergoing surgical debridement with a diagnosis of FG or NSTI were identified using ICD-10 codes. Outcomes included in-hospital mortality, length of stay (LOS), hospital costs, and home discharge rates. Multivariable regression analyses adjusted for patient demographics, comorbidities, and hospital characteristics.

**Results:**

A total of 5,007 FG and 24,782 non-perineal NSTI patients were identified. Crude in-hospital mortality rates were 5.8% for FG and 5.4% for non-perineal NSTIs, with stable trends observed over five years. After adjustment, no significant difference in mortality was observed (adjusted odds ratio [aOR]: 1.04; 95% CI: 0.90–1.20). However, FG was associated with longer LOS (adjusted mean difference: 1.99 days; 95% CI: 1.53–2.46) and higher hospital costs ($37,809 higher; 95% CI: $29,540–$46,077). Home discharge rates were similar between groups (aOR: 0.97; 95% CI: 0.89–1.05).

**Conclusion:**

Despite similar mortality rates, FG hospitalizations were associated with increased LOS and higher costs compared to non-perineal NSTIs. These findings suggest potential nationwide disparities in FG care quality, particularly outside specialized referral centers. Further research is needed to understand if standardized care pathways tailored to FG may optimize management and reduce resource utilization.

**Disclosures:**

All Authors: No reported disclosures

